# New insight into the selective photocatalytic oxidation of RhB through a strategy of modulating radical generation†

**DOI:** 10.1039/c8ra01810c

**Published:** 2018-04-11

**Authors:** Huijun Liang, Shengnan Liu, Hucheng Zhang, Xiaobing Wang, Jianji Wang

**Affiliations:** Collaborative Innovation Center of Henan Province for Green Manufacturing of Fine Chemicals, Key Laboratory of Green Chemical Media and Reactions, Ministry of Education, School of Chemistry and Chemical Engineering, Henan Normal University Xinxiang Henan 453007 P. R. China hzhang@htu.edu.cn jwang@henannu.edu.cn; College of Chemistry and Chemical Engineering, Xinxiang University Xinxiang Henan 453003 P. R. China

## Abstract

Rhodamine B (RhB) has often been used as a model pollutant, but its photocatalytic mechanism is still controversial. Herein, Ag NPs were sandwiched between CdS QDs and amorphous-TiO_2_ (a-TiO_2_) with the intent to build a CdS/Ag/a-TiO_2_ catalyst with highly selective oxidation activity. When rhodamine B (RhB) was used as the model organic compound, the CdS/Ag/a-TiO_2_ composite can not only modulate radical generation but also improve the conversion ratio of RhB to rhodamine 110 (Rh-110) to as high as 82% at 80 min during the visible-light irradiation. A series of the radical scavenging experiments revealed that CdS/Ag/a-TiO_2_ composites could modulate the effects of hydroxyl radicals (·OH) and superoxide anion radicals (·O_2_^−^) at different reaction stages so that the overoxidation of RhB and Rh-110 were repressed. Therefore, the transient state protection mechanism of selective oxidation of RhB was proposed to explain the reaction selectivity for Rh-110. Although the effects of both ·O_2_^−^ and ·OH are important during the photocatalytic selective oxidation of RhB, it is shown that the selective oxidation of RhB would be performed when the effect of ·O_2_^−^ is bigger than the ·OH, if not, RhB would be oxidized unselectively. Meanwhile, this may provide a new strategy for modulating radical generation in the photocatalysis of water phases.

## Abstract 2

A new photocatalytic mechanism of RhB was proposed according to the effect of reactive radical under different light irradiation.

## Graphical abstract

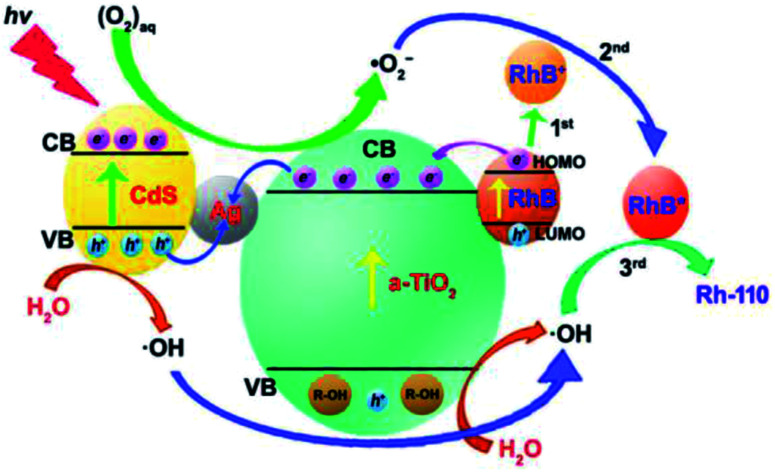

## 1. Introduction

As a model organic compound, rhodamine B (RhB) is often used for examining the activities of photocatalysts,^1,2^ Up to now, there have been two views on the photocatalytic degradation mechanism of RhB, one is chemisorption of functional groups, and the other is photosensitized degradation.^3–5^ Nevertheless, the two views both assumed that the *N*-deethylation of RhB would compete with the destruction of conjugated xanthene structure resulting in the uncomplete (selective oxidation) or complete degradation of RhB.^3,4,6–8^ However, according to the two photocatalytic degradation mechanisms, it is still difficult to control the oxidation process of RhB and obtain desired products (*e.g.* rhodamine 110, Rh-110).^9^

Heterogeneous photocatalysis of semiconducting metal oxides has been widely studied since Fujishima and Honda found the photoelectrochemistry of TiO_2_.^10^ So far, photocatalytic oxidation with O_2_ has been extensively used for unselectively obtaining the complete mineralization of a variety of pollutants in gaseous and aqueous phases because of the milder reaction conditions, ease of scaling up, environmentally friendly properties, and relatively high economic efficiency.^11–15^ Beyond that, the selective photocatalytic oxidation of pollutants or organics have also attracted attention in recent years because it can also produce high-valued chemicals,^11,16,17^ such as the selective oxidation of aromatic alcohols,^11,17–20^ aliphatic alcohols,^21–25^ alkenes and alkanes,^26^ amines,^14,27^ isoeugenol^28^ and so on.

However, it is well known that the photogenerated electrons (e^−^) and holes (h^+^) could react with dissolved oxygen [(O_2_)_aq_] or hydroxyl (–OH) on the surface of photocatalysts to produce various reactive oxygen species (ROS) during the reactions, such as hydroxyl radicals (·OH), superoxide anion radicals (·O_2_^−^), and O_2_^−^ protonation radicals (·OOH), and so on.^4,29^ For example, when the TiO_2_ is used as photocatalyst under ultraviolet (UV) light irradiation, the formations of ROS can be described as follow:^30–34^

1 TiO_2_ + *hv* → e^−^ + h^+^

2 (O_2_)_aq_ + e^−^ → ·O_2_^−^

3 h^+^ + H_2_O → H^+^ + ·OH

4 h^+^ + OH^−^ ⇌ ·OH

5 ·O_2_^−^ + H^+^ → ·OOH

6 ·OOH + ·O_2_^−^ + H^+^ → O_2_ + H_2_O_2_

7 H_2_O_2_ + ·O_2_^−^ → ·OH + OH^−^ + O_2_

This suggests that the photogenerated e^−^ and h^+^ can be converted into the different ROS. In fact, the nonselective auto-oxidations of ROS involve in many parallel oxidation pathways during photocatalytic oxidations,^35^ the photocatalytic selectivity of desired products is usually very low when the produces or substrates are seriously oxidized by these ROS in the water phase.^9,27,36,37^ However, many reports have demonstrated that ROS have different oxidation potential, such as 2.80 or 2.7 V for ·OH,^29,33^ 1.76 V for O_2_ or H_2_O_2_,^6^ and −0.33 V for ·O_2_^−^,^29^ indicating that ROS possess the different photocatalytic oxidation activity, hereinto, ·OH is the highest one according to the oxidation potential. Therefore, it is possible to perform the selective oxidation of RhB and improve the conversion ratio towards the Rh-110 through the decrease of ·OH during the photocatalytic oxidations. However, to the best of our knowledge, the mediating strategies for the selective generation of ROS are very few in the previous literature, and one of the main challenges is difficult to construct suitable framework for the blocks of photocatalysts.^11,21^

CdS quantum dots (QDs) respond sensitively to visible (Vis) light,^19^ and amorphous TiO_2_ (a-TiO_2_) efficiently captures h^+^.^38,39^ As the photogenerated h^+^ is directly trapped by a-TiO_2_, the ·OH radicals of higher oxidation activity would be decreased according to equation (eqn (3) and (4)), further, the photoinduced stability of CdS QDs and the conversion ratio of selective photooxidation would be improved during the photocatalytic oxidation.^40^ Therefore, it is expected that CdS/a-TiO_2_ composite could be more favorable for enhancing the effect of·O_2_^−^ rather than the ·OH with higher oxidation activity in the photooxidation.

However, the spectral response range of CdS QDs is relatively narrow,^41^ and a-TiO_2_ in nature has the short range ordered structure and the high defect density.^38,42^ These factors are very detrimental to the photocatalytic activity of CdS/a-TiO_2_ composites. It is well known that the localized surface plasmon resonance (LSPR) of noble metal nanoparticles (NPs) can induce a local electric field, increase the photocatalytic activity of a-TiO_2_, and extend the light absorption range of CdS/a-TiO_2_.^43^ Therefore, CdS/a-TiO_2_ composites containing noble metal NPs could be preferential catalysts for increasing the effect of ·O_2_^−^ and the conversion ratio of Rh-110.

In this work, the hydrosoluble CdS quantum dots (QDs) and Ag nanoparticles were loaded on a-TiO_2_ with high specific surface area, and respectively to prepare CdS/a-TiO_2_, CdS/Ag/a-TiO_2_ and CdS/Ag/c-TiO_2_ composites. The selective photooxidation activities of catalysts were evaluated by the yield of Rh-110. It is shown that CdS/a-TiO_2_ and CdS/Ag/a-TiO_2_ composites all exhibit the catalytic activity for the selective oxidation of rhodamine B under Vis-light irradiation. The conversion ratio of CdS/a-TiO_2_ is only about 9.7% in 100 min, however, the conversion ratio of CdS/Ag/a-TiO_2_ can reach to 82.4% in 80 min. Compare with our previous report, Ag/a-TiO_2_ (which was labeled Ag/TiO_2_–I in previous work) also has the selective oxidation performance, but the conversion ratio is only 23.8% in 180 min.^9^ In order to insight into the roles played by ·O_2_^−^, ·OH, and h^+^, the scavengers of benzoquinone (BQ), isopropanol (IPA), and triethanolamine (TEOA) were used respectively to remove ·O_2_^−^, ·OH, and h^+^ during the photocatalytic reaction. The scavenging experiments confirm that the ROS can be modulated by CdS/Ag/a-TiO_2_ composite during the photocatalytic reaction, and the conversion ratio of Rh-100 can be improved by the rational design of catalysts, so that the photocatalytic degradation mechanisms of RhB can be explained by the transient state protection mechanism of RhB.

## 2. Experimental section

Cadmium chloride (CdCl_2_·2.5H_2_O, AR) and absolute ethanol were purchased from Beijing Chemical Works (China). Ethylene glycol (EG, HOCH_2_CH_2_OH, AR) and tetrabutyl titanate (TBT, 98%) were purchased from Sinopharm Chemical Reagent Co. Ltd. Acetic acid (CH_3_COOH, AR) was purchased from Tianjin Deen Chemical Reagent Co. Ltd. Silver nitrate (AgNO_3_, AR) and thioacetamide (TAA) were purchased from Aladdin. RhB (AR) was purchased from Tianjin Kemiou Chemical Reagent Co., Ltd.

### 2.1 Synthesis of CdS QDs and a-TiO_2_

In a typical procedure, 2.08 g of CdCl_2_·2.5H_2_O and 0.75 g of TAA were respectively dissolved into 100 mL absolute ethanol by means of ultrasonication, then two solutions were mixed with the ultrasonication for 10 min and let it stand for 4 h to form the CdS precursor at room temperature.

The as-prepared CdS precursor was added into 300 mL deionized water, and then heated to 40 °C for 8 h under magnetic stirring. The colour of solution changes from colorless to yellow. The precipitate was separated by centrifugation at 20 000 rpm, and washed with deionized water and absolute ethanol several times, and finally dried in vacuum at 60 °C to obtain yellow hydrosoluble CdS QDs.

a-TiO_2_ was synthesized by the processes as reported in our previous work,^44^ and also the details can be found in ESI S1.†

### 2.2 Synthesis of CdS/a-TiO_2_ and CdS/Ag/a-TiO_2_ composites

2 g a-TiO_2_ was dispersed into 300 mL deionized water with the ultrasonication for 30 min, and then irradiated by UV-light source for 2 h. The color of suspension evolved gradually from white to black blue. After UV-light source was shut down, 50 mL aqueous solution containing 0.6 g of CdS QDs was added into the a-TiO_2_ suspension under vigorously magnetic stirring for 1 h. Then the precipitate was separated by centrifugation at 10 000 rpm, and washed with deionized water and absolute ethanol several times, and finally dried in vacuum at 40 °C to obtain CdS/a-TiO_2_ composite.

After UV-light source was shut down, similarly, 20 mL AgNO_3_ aqueous solution (19.4 mmol L^−1^) was mixed into the black blue a-TiO_2_ under vigorously magnetic stirring for 1 h to obtain gray Ag/a-TiO_2_ composite. Then, 50 mL aqueous solution containing 0.6 g of CdS QDs was added into under stirring for 1 h. After this, the precipitate was separated, washed, and dried following the same processes as mentioned above to harvest CdS/Ag/a-TiO_2_ composite.

CdS/Ag/c-TiO_2_ was prepared through annealing CdS/Ag/a-TiO_2_ at 300 °C for 4 h for comparison.

### 2.3 Photochemical reactor and photocatalytic experiments

The photochemical reactor system was consisted of a 30 mL quartz tube and a cylindrical water-cooled jacket quartz cell. A 500 W Xe-lamp or 500 W high-pressure mercury lamp was placed inside the quartz cell, which was respectively used as the Vis and UV-light source. The optical path length was 10 cm. The cylindrical water-cooled jacket quartz cell was kept at 15 ± 2 °C by an external cooling jacket with recycled water.

The photocatalytic degradation of 5 mg L^−1^ RhB was used to evaluate the selective oxidation activities of as-synthesized samples and the effect of ·O_2_^−^, ·OH, and h^+^. Typical, 25 mL RhB solution and 0.05 g catalysts were dispersed into 30 mL quartz tube under magnetically stirred and open to air, and were held in dark for 30 min to reach adsorption–desorption equilibrium. After irradiated by light for a certain time interval, 2 mL reaction mixture was centrifuged to remove the photocatalyst, and the degradation of RhB was evaluated by the intensity of adsorption peak at 554 nm. The percentages of degradation is calculated as *C*/*C*_0_ × 100%, here, *C* is the intensity of the RhB solution at each irradiated time interval, and *C*_0_ is the intensity of the initial concentration.

In order to simplify the calculation, the concentration of selective photooxidation product (Rh-110) was evaluated by the intensity of adsorption peak at 498 nm according to the molecular amount of converting RhB into Rh-110 (eqn (8)), and correspondingly the conversion ratio is also calculated as *C*/*C*_0_ × 100%. Especially, the conversion ratio was marked by blue pentagrams at the degradation efficiency curves only when the Rh-110 was generated. It means that the conversion ratio increase with increasing the concentration of Rh-110, but the degradation efficiency decrease.

In the same experimental conditions, a certain amount of benzoquinone (BQ), isopropanol (IPA), and triethanolamine (TEOA) were respectively added into the photocatalytic degradation system of RhB to evaluate the effect of ·O_2_^−^, ·OH, and h^+^ according to the degradation percentage of RhB.

### 2.4 Structural characterizations

The as-prepared samples were characterized by the transmission electron microscope (TEM, JEOL JEM-2100), X-ray diffraction (XRD, Bruker advance-D8), X-ray photoelectron spectroscopy (XPS, VG Scientific ESCALAB MKII), and Brunauer–Emmet–Teller (BET) method and the Barrett–Joyner–Halenda (BJH, Micromeritics Instruments ASAP 2020). Their properties were measured by the UV-Vis diffused reflectance spectra (DRS, Lambda 950, PerkinElmer Inc, America, and BaSO_4_ was used as a reflectance standard.), photoluminescence spectrophotometer (PL, HITACHI FP-6500 spectrophotometer), and UV-Vis spectrophotometry (TU-1900, Beijing). The elemental compositions of samples were investigated by the energy-dispersive X-ray spectroscopy (EDS) attached to the field emission scanning electron microscopy (FESEM, ZEISS SUPRA-40 VP).

## 3. Results and discussions

### 3.1 Synthesis and characterizations of composites

**Fig. 1:**
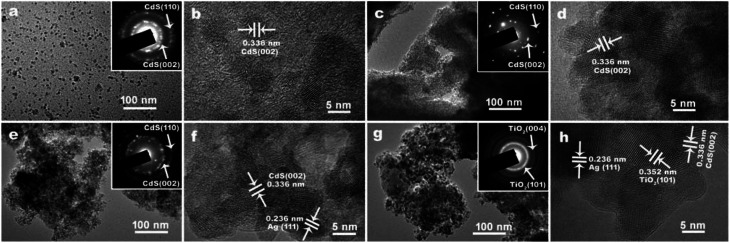
The TEM and HRTEM images of as-prepared samples. (a) and (b) CdS QDs, (c) and (d) CdS/a-TiO_2_, (e) and (f) CdS/Ag/a-TiO_2_, (g) and (h) CdS/Ag/c-TiO_2_. The insets are the ring patterns of the selected area electron diffraction (SAED) images.

CdS QDs are usually synthesized by the organic phase or aqueous phase routes. By contrast, aqueous phase syntheses of CdS QDs are simpler and easily scaled up, but the resulting QDs often exhibit lower crystallinity and quantum yields.^45,46^ Here, the uniform hydrosoluble CdS QDs were synthesized by a facile hydrolysis method without surfactant. As shown from TEM images in Fig. 1a and b, the as-prepared CdS QDs have good dispersity and uniformity with the mean size about 5.4 ± 0.2 nm (Fig. S1a†). The lattice spacing of 0.336 nm corresponding to the (002) plan of CdS in high-resolution transmission electron micro (HRTEM) and the pattern of the selected area electron diffraction (SAED) confirm the resulting QDs possess good crystallinity.

When the CdS QDs were loaded on the surface of a-TiO_2,_ the images in Fig. 1c and d show the relatively uniform distribution of CdS QDs with almost unchanged size of 5.6 ± 0.2 nm (Fig. S1b†). As reported in our previous work, the light chemical reduction in aqueous AgNO_3_ solution can induce the growth of Ag NPs on the a-TiO_2_ to produce Ag/a-TiO_2_ composite.^9^ It suggests that the Ag NPs on Ag/a-TiO_2_ composite can provide the good scaffolds to anchor CdS QDs through S–Ag chemical interactions. As a result, CdS/Ag/a-TiO_2_ composite was facilely prepared by mixing CdS QDs with Ag/a-TiO_2_ suspension. Despite the stacking of CdS QDs on Ag NPs, the metal occurrence in CdS/Ag/a-TiO_2_ composite could still be identifiable from the lattice spacing of Ag (111) crystal plane (corresponding to 0.236 nm in Fig. 1f) and a few Ag Nps with big particle size (Fig. S2†). As shown in Fig. S2,† some representative Ag NPs show that CdS QDs are loaded on the Ag NPs (white arrows), and Ag NPs located at between CdS QDs and a-TiO_2_. The architecture was further confirmed by the similar distribution of Cd and Ag elements on a-TiO_2_ from the element mapping images of CdS/Ag/a-TiO_2_ (Fig. S3†). After annealing CdS/Ag/a-TiO_2_, however, the geometric arrangement in the composite could be disarrayed to turn into the CdS/Ag/crystalline TiO_2_ (CdS/Ag/c-TiO_2_) composite, and hence Ag NPs were exposed on TiO_2_ and can be clearly observed from Fig. 1g and h. Simultaneously, the SAED images and the lattice spacing of 0.352 nm corresponding to the (101) plan of TiO_2_ verify the transformation of a-TiO_2_ to crystalline TiO_2_ (c-TiO_2_) after the annealing treatment of CdS/Ag/a-TiO_2_.

**Fig. 2:**
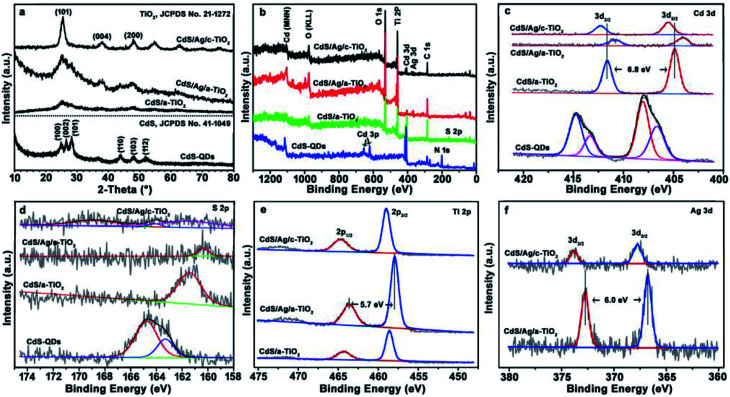
The XRD patterns and XPS spectra of the as-prepared samples. (a) XRD patterns, (b) survey spectra, and the high-resolution XPS spectra of (c) Cd 3d, (d) S 2p; (e) Ti 2p; (f) Ag 3d.

The phase structure and composition of as-prepared samples were determined by XRD patterns and XPS spectra. As shown in Fig. 2a, the diffraction peaks show that CdS QDs represent a hexagonal phase (JCPDS card no. 41-1049), but the wide diffraction peaks of CdS/a-TiO_2_ and CdS/Ag/a-TiO_2_ composites result from the a-TiO_2_ due to the low content of CdS and Ag beyond the instrumental detection limit. After annealing CdS/Ag/a-TiO_2_, the diffraction pattern indicates that the anatase TiO_2_ occurs in CdS/Ag/c-TiO_2_ (JCPDS no. 21-1272).

The survey scan XPS spectra of as-prepared samples clearly show the occurrence of the expected Ti, O, Cd, S, C, and N elements in the respective composite (Fig. 2b and Table S1†). It is noted that Cd and S elements can be detected from all samples, but N element only from CdS QDs due to the presence of TAA ligands. The absence of N 1s peak in the survey spectra of CdS/a-TiO_2_, CdS/Ag/a-TiO_2_ and CdS/Ag/c-TiO_2_ indicates the remove of TAA ligands, and reflects that the strong interactions of CdS with a-TiO_2_ and Ag NPs cause the QDs almost “naked” in these composites.

The chemical states of Cd 3d, S 2p, Ti 2p, and Ag 3d in the as-prepared samples show distinct differences in their high-resolution XPS spectra (Fig. 2c–f), respectively. As shown in Fig. 2c, the XPS spectra of Cd 3d in CdS QDs shows that the Cd 3d_3/2_ and 3d_5/2_ peak can be divided into two peaks centered at ∼414.8/413.4 and ∼408.0/406.6 eV, and the peak separation of 6.8 eV corresponding to Cd^2+^. The peaks at 413.4/406.6 eV are ascribed to CdS, and the other at 414.8/408.0 eV are combined with TAA ligands.^47,48^ When the CdS QDs are loaded on a-TiO_2_, the Cd 3d_3/2_ and Cd 3d_5/2_ appears as a single peaks in agreement with the remove of TAA, and the strong interactions cause the peak shift to 411.6/404.8 eV. Furthermore, the Cd 3d peak is shifted to 410.9/404.1 eV in CdS/Ag/a-TiO_2_ due to the chemical bonding between S and Ag as the CdS QDs are loaded on Ag/a-TiO_2_, it means that the strong interactions between CdS QDs, Ag NPs and a-TiO_2_ cause the peak shift of Cd 3d.^49,50^ Correspondingly, the binding energy of S 2p exhibit the similar changes in pace with the enhanced interactions in the composites (Fig. 2d). By contrast, the Cd 3d peak in CdS/Ag/c-TiO_2_ reversely shifts to 412.3/405.5 eV, a binding energy more than that in CdS/a-TiO_2_, implying the collapse of stacking structure in CdS/Ag/c-TiO_2_ due to the annealing treatment and the weaker interactions between CdS and c-TiO_2_ in the resulting composite. Additionally, the architecture transformation of the three blockings during annealing can be verified from the high-resolution spectra of Ti and Ag elements: the Ti 2p peak shifts from 464.3/458.6 eV in CdS/a-TiO_2_ to 463.6/457.9 eV in CdS/Ag/a-TiO_2_, then to 464.7/459.0 eV in CdS/Ag/c-TiO_2_, the Ag 3d peak from 372.8/366.8 eV in CdS/Ag/a-TiO_2_ to 373.8/367.8 eV in CdS/Ag/c-TiO_2_ (Fig. 2e and f).^51,52^

**Fig. 3:**
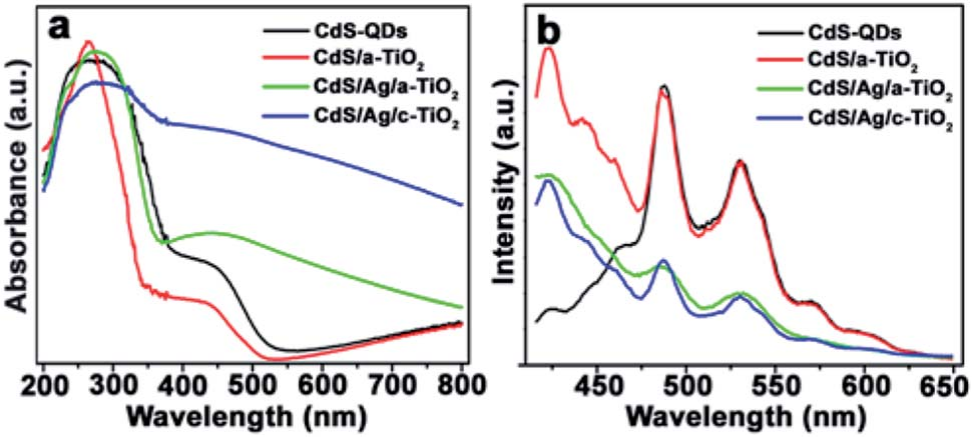
(a) UV-Vis DRS, and (b) PL spectra of as-prepared composites.

Fig. 3 shows several optical properties of as-prepared samples. The pristine CdS QDs and CdS/a-TiO_2_ exhibit a Vis-light absorption edge at about 450 nm (Fig. 3a), resulting from the intrinsic band-gap transition of electron from the valence band (VB) to the conduction band (CB) in CdS.^53^ When Ag NPs are added into CdS/a-TiO_2_, the synergistic effect of the LSPR with the light absorption of CdS QDs promotes the absorption of CdS/Ag/a-TiO_2_ and CdS/Ag/c-TiO_2_ composites extends over wider Vis-light region.^47,54,55^ The band gap (*E*_g_) of CdS QDs, CdS/a-TiO_2_, and CdS/Ag/a-TiO_2_ is respectively estimated to be 2.5, 3.1, and 2.7 eV by Tauc plot of (*Ahν*)^2^*vs. hν* (Fig. S4a†).^52,56,57^

The emission intensity of photoluminescence (PL) spectra demonstrates the concentration of charge carriers in the excited states of composites. As excited by 386 nm, the as-prepared CdS QDs exhibit two main emission peaks located at 487 and 531 nm (Fig. 3b) that originate from the excitonic recombination.^55^ The relative emission intensities of CdS hold strong in CdS/a-TiO_2_, but significantly are weaken in CdS/Ag/a-TiO_2_ and CdS/Ag/c-TiO_2_, and particularly in CdS/Ag/a-TiO_2_. This suggests that the introduction of Ag NPs between CdS and a-TiO_2_ can moderately restrain the combination of photogenerated holes and electrons,^58^ and is expected to modulate ROS and to perform selective photocatalysis. The excited state electron radioactive decay lifetime of samples can also confirm the inhibition of Ag in CdS/Ag/a-TiO_2_. As shown in Fig. S4b,† the calculated average lifetime of CdS QDs, CdS/a-TiO_2_, CdS/Ag/a-TiO_2_ and CdS/Ag/c-TiO_2_ are 3.5, 4.4, 4.3 and 4.9 ns, respectively. The lifetime of CdS/Ag/a-TiO_2_ is longer than the CdS QDs.

In addition, the specific surface area, pore volume and average pore size of the composites were measured by the nitrogen adsorption/desorption experiments (Fig. S5 and Table S2†). Among these composites, CdS/Ag/a-TiO_2_ with the medium pore size exhibits the largest BET surface area of 441 m^2^ g^−1^ and pore volume of 0.35 cm^3^ g^−1^, and can provide the optimal space for available photocatalystic reactions.

### 3.2 Selective photocatalytic oxidation of RhB

**Fig. 4:**
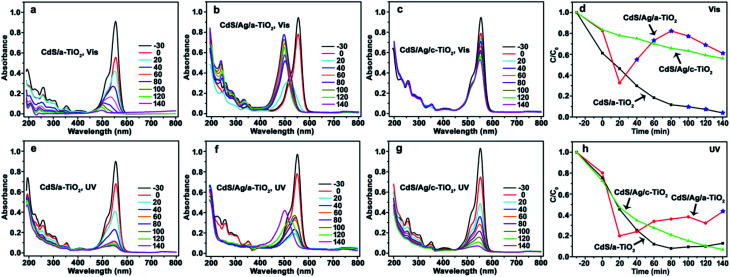
Evolution of UV-Vis spectrum and photocatalytic degradation efficiency of RhB using the different photocatalysts and irradiation light sources. (a) CdS/a-TiO_2_, (b) CdS/Ag/a-TiO_2_, (c) CdS/Ag/c-TiO_2_, (d) degradation efficiency under Vis-light; and (e) CdS/a-TiO_2_, (f) CdS/Ag/a-TiO_2_, (g) CdS/Ag/c-TiO_2_, (h) degradation efficiency under UV-light. The conversion ratio of Rh-110 was marked by the blue pentagrams at the corresponding degradation efficiency curves (d and h).

When CdS/a-TiO_2_, CdS/Ag/a-TiO_2_, and CdS/Ag/c-TiO_2_ were respectively used as photocatalysts, and correspondingly the changes of UV-Vis spectrum and degradation efficiency with time were recorded from the RhB aqueous solution during the photooxidation (Fig. 4). As far as Vis-light irradiation is concerned, the maximum absorption wavelength (*λ*_max_) of RhB solution in the presence of CdS/a-TiO_2_ blue-shifts from 554 to 498 nm (Fig. 4a), indicating the formation of Rh-110 and the selective photocatalytic oxidation toward RhB.^9^ Although the conversion ratio of Rh-110 is only about 9.7% at 100 min, CdS/a-TiO_2_ exhibits the highest degradation efficiency compared with the other photocatalysts (Fig. 4d). CdS/Ag/a-TiO_2_ represents the most efficient selective photocatalysis (Fig. 4b and d). The *λ*_max_ of RhB solution blue-shifts to 510 nm only in 20 min that is attributed to the photoisomerization of dye chromophores, an intermediate for the conversion from RhB to Rh-110.^1,2,4,9,33,59^ After 20 min, the peak intensity at *λ*_max_ of Rh-110 increases with irradiation time, and reaches the maximum (at 80 min) that almost equates to the peak intensity of RhB in the adsorption–desorption equilibrium solution (corresponding to the absorption peak intensity at 0 min in Fig. 4b). At this phase, the conversion ratio for Rh-110 is about 82%, subsequently, the peak intensity at 498 nm reduces gradually due to the degradation of Rh-110 with increasing irradiation time from 80 to 140 min. Unlike CdS/Ag/a-TiO_2_, however, no photocatalysis of the selective oxidation is observed from the CdS/Ag/c-TiO_2_ (Fig. 4c and d), and also its photocatalytic degradation efficiency is lower than CdS/a-TiO_2_. The experimental facts suggest that the special geometric arrangement in CdS/Ag/a-TiO_2_ is responsible for the selective photocatalytic conversion from RhB to Rh-110 with Vis-light irradiation.

When the RhB solution was degraded by UV-light irradiation, both CdS/a-TiO_2_ and CdS/Ag/c-TiO_2_ exhibit the high photocatalytic degradation efficiency (Fig. 4e, g and h), and the RhB solution almost completely become colorless in 140 min, but the selective oxidation product of Rh-110 was not detected from the photocatalysis. When the CdS/Ag/a-TiO_2_ is used as photocatalyst, by contrast, the blue-shift of *λ*_max_ for RhB solution can be observed, and the conversion ratio from RhB to Rh-110 is estimated to be 43% at 140 min (Fig. 4f and h), a value much less than that obtained from Vis-light irradiation. Therefore, CdS/Ag/a-TiO_2_ is a preferential photocatalyst for the selective oxidation through UV- and Vis-light treatment, and demonstrates the great selectivity and activity under Vis-light irradiation.

### 3.3 Modulating ROS and the photocatalytic mechanism of RhB



8

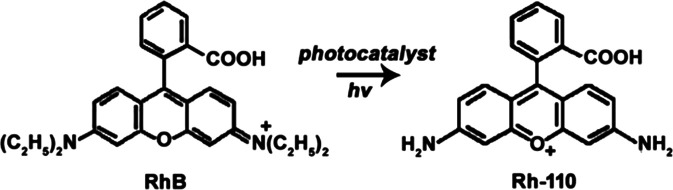




RhB as a model organic compound has been widely used to evaluate the photocatalytic degradation reaction because RhB could be not only completely degraded but also selectively photooxidized to produce Rh-110 (eqn (8)).^1,2,4,7,33^ In our previous report, we demonstrated that RhB could be selectively oxidized using Vis-light source and Ag/a-TiO_2_ photocatalyst to harvest Rh-110 with the conversion ratio of 23.8% in 180 min.^9^ Under three light-emitting diode (LED, 1 W, radiant wavelength of 455–460 nm), alternatively, it is shown that both Ag/a-TiO_2_ (Fig. S6a†) and Ag/c-TiO_2_ (Fig. S6b†) can act as photocatalyst for selective oxidation, and the conversion ratio for Rh-110 is 39% at 40 min using Ag/a-TiO_2_ and is 14% at 80 min using Ag/c-TiO_2_. Unfortunately, it is the low conversion ratio that limits the practical applications of the selective photocatalytic oxidations.

**Fig. 5:**
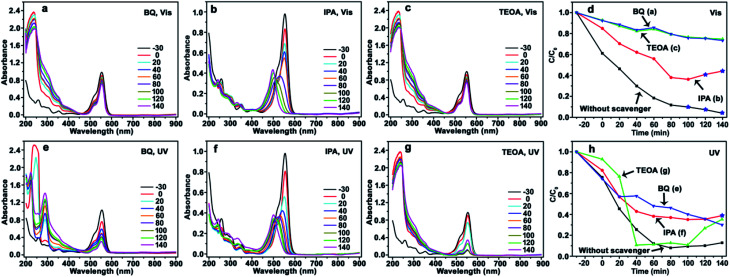
Evolution of UV-Vis spectrum and photocatalytic degradation efficiency of RhB using CdS/a-TiO_2_ photocatalyst. Scavengers: BQ (a), IPA (b), TEOA (c), and the corresponding degradation efficiency (d) under Vis-light; BQ (e), IPA (f), TEOA (g), and the corresponding degradation efficiency (h) under UV-light. The degradation efficiency curves without scavenger is inserted into (d) and (h) for comparison. The conversion ratio of Rh-110 was marked by the blue pentagrams at the corresponding degradation efficiency curves (d and h).

**Fig. 6:**
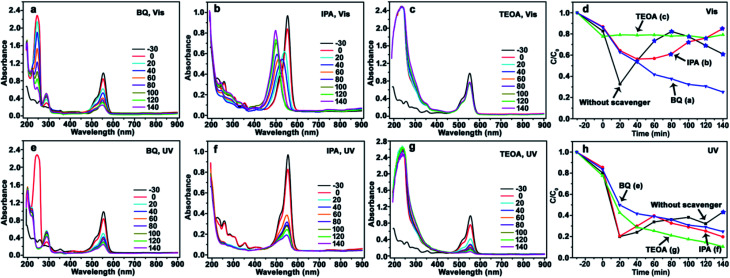
Evolution of UV-Vis spectrum and photocatalytic degradation efficiency of RhB using CdS/Ag/a-TiO_2_ photocatalyst. Scavengers: BQ (a), IPA (b), TEOA (c), and the corresponding degradation efficiency (d) under Vis-light; BQ (e), IPA (f), TEOA (g), and the corresponding degradation efficiency (h) under UV-light. The degradation efficiency curves without scavenger is inserted into (d) and (h) for comparison. The conversion ratio of Rh-110 was marked by the blue pentagrams at the corresponding degradation efficiency curves (d and h).

Herein, CdS/Ag/a-TiO_2_ not only exhibits the wider light absorption range, but also the much higher selective oxidation activity with the conversion ratio of 82% for Rh-110 under Vis-light irradiation. Based on eqn (1) to (7), it is believed that CdS/Ag/a-TiO_2_ could promote the generation of ·O_2_^−^, but depress the increase of ·OH. In order to insight into the controlling mechanisms of selective photocatalytic oxidation of RhB, benzoquinone (BQ), isopropanol (IPA), and triethanolamine (TEOA) are used as scavengers respectively to remove ·O_2_^−^, ·OH, and h^+^ under the different light-sources,^60,61^ and the UV-Vis adsorption spectra of RhB solution and degradation efficiencies are shown in Fig. 5, 6 and 8 when the CdS/a-TiO_2_, CdS/Ag/a-TiO_2_, and CdS/Ag/c-TiO_2_ were respectively used as photocatalyst.

The photocatalysis of CdS/a-TiO_2_ in the presence of scavengers is shown in Fig. 5. After BQ and TEOA scavenge ·O_2_^−^ and h^+^, the obvious blue-shift of *λ*_max_ is not observed from RhB solution under Vis (Fig. 5a and c) and UV-light (Fig. 5e and g) irradiation, and the photocatalytic degradation rate is remarkably decreased under Vis-light irradiation (Fig. 5d), suggesting that both ·O_2_^−^ and h^+^ are important for the selective photocatalytic conversion of RhB to Rh-110. In addition, as shown in Fig. 5d, the effects of h^+^ is almost consistent with the ·O_2_^−^ (green and blue line), it suggesting that the photooxidized activities of h^+^ and ·O_2_^−^ in using CdS/a-TiO_2_ catalyst are almost identical under Vis-light irradiation.

In fact, according to the eqn (3) to (7), we propose that the effects of h^+^ can be considered together with the·O_2_^−^ and ·OH, that is, the effects of ·O_2_^−^ and ·OH are considered mainly. Although h^+^ itself has certain photooxidized activity (which is decided by the valence band position of materials.), the effects of h^+^ can be final converted into the ·O_2_^−^ and ·OH according to the eqn (3) to (7). It means that the effects of ·O_2_^−^ and ·OH are simultaneous changed with the h^+^. Therefore, in order to simplify discussion, the effects (or amount) of ·O_2_^−^ and ·OH are considered mainly in this work. Moreover, according to the simultaneous changed of ·O_2_^−^ and ·OH with the h^+^, the curve overlapping of ·O_2_^−^ (Fig. 5d, BQ) and h^+^ (Fig. 5d, TEOA) is easy explained. Under Vis-light irradiation, a-TiO_2_ is not excited by the Vis-light, the photogenerated e^−^ and h^+^ is mainly originated from CdS QDs. The photogenerated e^−^ on the surface of CdS QDs can be scavenged by surface-adsorbed oxygen (eqn (2)), and h^+^ can be left in the CdS QDs.^62^ When the h^+^ is scavenged, the amount of ·O_2_^−^ and ·OH also simultaneous decrease, so that the curves of ·O_2_^−^ and h^+^ are accidentally overlapped.

Using IPA as scavenger to remove ·OH, by contrast, the *λ*_max_ of RhB solution exhibit obvious blue-shifts under both Vis (Fig. 5b) and UV-light (Fig. 5f) irradiation, and the conversion ratio of Rh-110 can reach to 44% at 140 min under Vis-light irradiation. Compared with the conversion ratio of 9.7% for CdS/a-TiO_2_ (Fig. 4a and d), the scavenging of ·OH further improve the conversion ratio of Rh-110, suggesting that the scavenging of ·OH with the high photooxidized activity decreases the overoxidation of product (Rh-110) and substance (RhB) due to the high photooxidized activity of ·OH.

According to the photocatalytic degradation curve of RhB (Fig. 5d and h), one rule of selective photocatalytic oxidation can also be obtained, that is, the selective photocatalytic oxidation of RhB can be performed when the effect (or amount) of·O_2_^−^ is bigger than the ·OH in the reaction system, if not, RhB will be complete oxidized. As shown in Fig. 5d, the effect of ·O_2_^−^ is bigger than the ·OH under Vis-light irradiation, so that the selective photocatalytic oxidation of RhB can be performed by the pure CdS/a-TiO_2_ (Fig. 4a).

However, as shown in Fig. 4e, the blue-shift of *λ*_max_ is not observed. It can be attributed to the overoxidation of substance (RhB) at the initial stage of UV-light irradiation. Because the effect of ·OH is bigger than the ·O_2_^−^ before 20 min, a large number of RhB could have been complete oxidized at the initial stage, and Rh-110 cannot be obtained. In order to test this speculation, we performed a controlled experiment at same condition except for changing the concentration of RhB to 10 mg L^−1^. As shown in Fig. S7(see ESI†), Rh-110 is generated at 330 min. Therefore, the scavenging of ·OH is benefit to avoid the overoxidation of substance or product and improve the conversion ratio of product. As shown in Fig. 5f, when the ·OH is scavenged, the Rh-110 is obtained again at 140 min.

The similar phenomenon can be observed in the photocatalysis of CdS/Ag/a-TiO_2_. As shown in Fig. 6, the blue-shift of *λ*_max_ is not observed from RhB solution after scavenging ·O_2_^−^ (Fig. 6a and e) and h^+^ (Fig. 6c and g). In contrast, the *λ*_max_ of RhB solution exhibit obvious blue-shift under Vis-light irradiation (Fig. 6b). However, the blue-shift of *λ*_max_ disappear when the ·OH is scavenged UV-light irradiation (Fig. 6f). This phenomenon can be explained easily by the transient state protection mechanism, and obtaining the other rule of selective photocatalytic oxidation. That is, both ·O_2_^−^ and ·OH are important for the selective photocatalytic conversion of RhB to Rh-110, the conversion ratio of Rh-110 can be improved when the effect (or amount) of ·OH is suitable increased at the later stage.

In the previous work, we proposed that the effect of ·O_2_^−^ is more important than the ·OH during the process of forming Rh-110, because the conjugated xanthene structure of RhB could react with ·O_2_^−^ to first form a transient state.^9^ This transient state can protect the conjugated xanthene structure of RhB from damage, so that only *N*-deethylation reaction of RhB can be performed by ·OH to form Rh-110. In contrast, when the effect of ·OH is bigger than ·O_2_^−^, the conjugated xanthene structure of RhB is first damaged by the ·OH, so that the transient state of conjugated xanthene structure of RhB is not produced, Rh-110 can not be obtained. It suggests that the selective photocatalytic oxidation of RhB derives from the combined effect of ·OH and ·O_2_^−^.

**Fig. 7:**
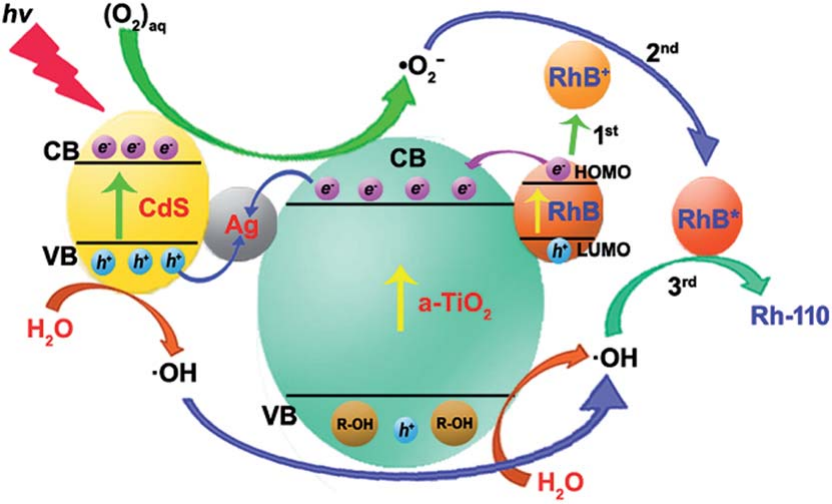
Schematic diagram of the possible process for CdS/Ag/a-TiO_2_ composites photocatalytic degrading RhB under light irradiation.

Therefore, the selective oxidation mechanism of CdS/Ag/a-TiO_2_ can be illustrated in Fig. 7. Under Vis-light irradiation, CdS/Ag/a-TiO_2_ can provide a large number of the photogenerated e^−^ and h^+^ due to the valence band (VB) electrons excitation of CdS and the formation of Z-scheme.^63,64^ Because the formation of Z-scheme, the combination of e^−^ and h^+^ should be suppressed during the complete photocatalysis oxidation (or unselective oxidation). However, the selective oxidation of RhB is the uncomplete photocatalysis oxidation process, we hope to reduce the effect of ·OH and obtain more ·O_2_^−^, the combination of e^−^ (derived from a-TiO_2_ and RhB) and h^+^ (derived from CdS QDs) at Ag NPs should be encouraged because it can improve the effect of ·O_2_^−^ and obtain the high conversion ratio. Meanwhile, RhB lost e^−^ of the highest occupied molecular orbital (HOMO) to RhB^+^ with positively charged (Fig. 7, 1st) due to the photosensitized of RhB. The result is that CdS/Ag/a-TiO_2_ shows the rich e^−^ state. The photoisomerization of RhB chromophores can be seen as a feature of this phenomenon (Fig. 4d, red line).^9,59^ Under UV-light irradiation, a-TiO_2_ is excited and h^+^ is captured, so that CdS/Ag/a-TiO_2_ also shows the rich e^−^ state (Fig. 4 h, red line). According to eqn (1) to (7), a large number of ·O_2_^−^ and a few ·OH are formed. Due to the electrostatic interaction, the reaction between ·O_2_^−^ and RhB^+^ is easy to form a transient state of RhB (RhB*, Fig. 7, 2nd) rather than the oxidation of RhB, then RhB* is oxidized by ·OH (Fig. 7, 3rd) to form Rh-110.

The transient state protection mechanism can be further confirmed by the scavenging experiments, especially the scavenging of ·OH. Under Vis-light irradiation, as shown in Fig. 6b, the *λ*_max_ of RhB solution gradually shifts from 554 nm (RhB) to 498 nm (Rh-110). Compared to pure CdS/Ag/a-TiO_2_ (Fig. 4b), the shifts rate is slowly. The reason is that *N*-deethylation reaction of RhB* is also slowly when the ·OH is scavenged, so that the serious overoxidation of RhB or Rh-110 is avoided, finally, the conversion ratio of Rh-110 reach to 85% at 140 min (Fig. 6b and d).

However, under UV-light irradiation, although a large number of RhB* is generated, the effect (amount) of ·OH is always weak during photocatalytic oxidation (Fig. 6h, red line), so the Rh-110 can be measured until 140 min (Fig. 4f). The reason is that *N*-deethylation reaction of RhB* is more slowly when the ·OH is scavenged (Fig. 6f).

It means that the effect of ·OH cannot be neglected during the selective photocatalytic oxidation of RhB. When the effect of ·OH is eliminated, the *N*-deethylation reaction of RhB* can not be performed so that the selective photocatalytic oxidation of RhB disappears. Therefore, the rational controls of various ROS are an essential prerequisite for highly selective oxidation reactions.^35^ CdS/Ag/a-TiO_2_ certainly control radical generation during the selective photocatalytic oxidation of RhB.

**Fig. 8:**
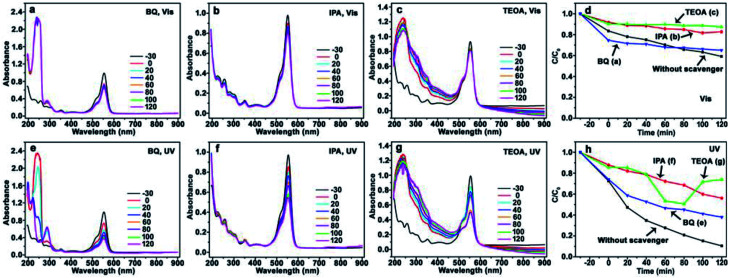
Evolution of UV-Vis spectrum and photocatalytic degradation efficiency of RhB using CdS/Ag/c-TiO_2_ photocatalyst. Scavengers: BQ (a), IPA (b), TEOA (c), and the corresponding degradation efficiency (d) under Vis-light; BQ (e), IPA (f), TEOA (g), and the corresponding degradation efficiency (h) under UV-light. The degradation efficiency curves without scavenger is inserted into (d) and (h) for comparison.

When the effect of ·OH is bigger than the ·O_2_^−^, the blue-shift of *λ*_max_ disappear, suggesting that RhB can not be selective oxidized. As shown in Fig. 8, after scavenging ·O_2_^−^ (Fig. 8a and e), ·OH (Fig. 8b and f), and h^+^ (Fig. 8c and g), the blue-shift of *λ*_max_ is not observed under both Vis- and UV-light irradiation. The photocatalytic degradation efficiency curves (Fig. 8d and h) demonstrate that the effect of ·OH is always bigger than the ·O_2_^−^. Because c-TiO_2_ can not capture h^+^, the amount of e^−^ is almost equate with the h^+^, the amount (effect) of ·OH increases. This result further confirms that the selective oxidation of RhB can be explained by the transient state protection mechanism.

## 4. Conclusion

In summary, we designed and synthesized a CdS/Ag/a-TiO_2_ composite with highly selective oxidation activity for the photocatalytic degradation of RhB. Due to the light-responding nature of CdS QDs and the LSPR effect of Ag NPs, CdS/Ag/a-TiO_2_ composites can not only improve the conversion ratio of RhB to Rh-110 in 82% at 80 min but also adjust the effect of ROS under Vis-light irradiation. A serial of the radical scavenging experiments confirm that CdS/Ag/a-TiO_2_ composite exhibits the good photocatalytic selective oxidation activity, because CdS/Ag/a-TiO_2_ composite can modulate the effect of ·O_2_^−^ and ·OH during the photocatalytic oxidation of RhB. Meanwhile, this work also further endorses the transient state protection mechanism of RhB as reported in previous work, that is, the effect of ·O_2_^−^ is more important than the ·OH in the forming process of Rh-110, because ·O_2_^−^ can react with RhB to form the transient state of conjugated xanthene structure of RhB and avoid the serious overoxidation toward RhB or Rh-110, but the effect of ·OH can not be neglected. Therefore, this may provide a new strategy for modulating radical generation in the photocatalysis of water phase.

## Conflicts of interest

There are no conflicts to declare.

## Supplementary Material

RA-008-C8RA01810C-s001
