# Supplementary material for: New insight into the selective photocatalytic oxidation of RhB through a strategy of modulating radical generation
Source: RSC Adv. 2018 Apr 11;8(24):13625–34. doi: 10.1039/c8ra01810c (PMC9079813; doi:10.1039/c8ra01810c)
Supplement: RA-008-C8RA01810C-s001 [file RA-008-C8RA01810C-s001.pdf]

Electronic Supplementary Material (ESI) for RSC Advances.
This journal is © The Royal Society of Chemistry 2018

Supplementary Information

New insight into the selective photocatalytic oxidation of RhB through a strategy of modulating radical generation

Huijun Liang,[a,b] *Shengnan Liu,*[a] *Hucheng Zhang,*[*,a] *Xiaobing Wang*[a] *and Jianji Wang*[*,a]

[a] Collaborative Innovation Center of Henan Province for Green Manufacturing of Fine Chemicals, Key Laboratory of Green Chemical Media and Reactions, Ministry of Education, School of Chemistry and Chemical Engineering, Henan Normal University, Xinxiang, Henan 453007, P. R. China

[b] College of Chemistry and Chemical Engineering, Xinxiang University, Xinxiang, Henan 453003, P. R. China

S1. ***The synthesis of TiO_2 precursors***

TiO_2 precursors were synthesized by an acid-etching method in our previous report.[1] In a typical procedure, 15 mL TBT was added to EG (100 mL) under vigorously magnetic stirring for 30 min. Then the solution was heated at 164 °C for 2 hours under magnetic stirring. Finally, the white precipitate was obtained at the bottom of the flask. After cooling down to room temperature, 0.5 mL acetic acid solution (3 M) was added to the flask. Reheating at 107 °C for 2h, the white precipitate was obtained through centrifuging and washing with deionized water and absolute ethanol. The as-prepared sample was dried at 100 °C for 10 h and denoted as a-TiO_2 precursors.

The photochemical reactor was consisted of a cylindrical water-cooled jacket

quartz cell. The light source was placed inside the quartz cell, and the optical path length was about 1 cm. A 500 W Xe-lamp and 500 W high-pressure mercury lamp was used as the visible and UV light source (main output 365 nm), respectively.

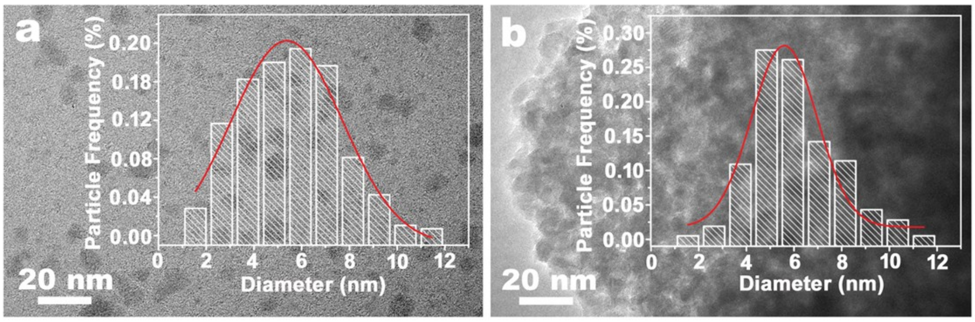

Fig. S1 The TEM images and the size distribution of as-prepared sample. (a) CdS-QDs, (b) CdS/a-TiO_2. The mean size of CdS-QDs is 5.4±0.2 nm (Fig. S1a), the mean size of CdS is 5.6±0.2 nm in the CdS/a-TiO_2 (Fig. S1b).

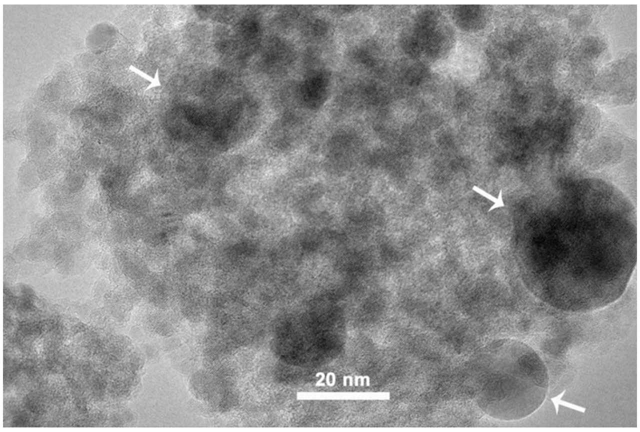

Fig. S2 The HRTEM images of as-prepared CdS/Ag/a-TiO_2. Typically Ag NPs is marked by the white arrows.

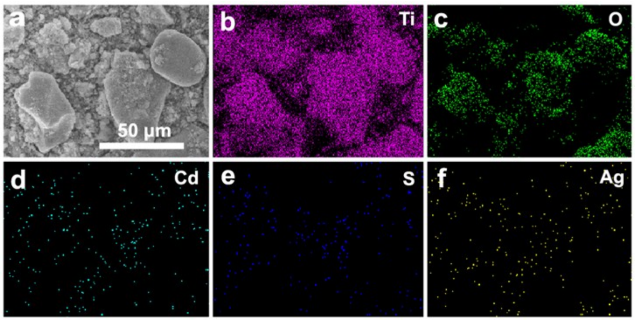

Fig. S3 The FESEM and elemental mapping images of as-prepared CdS/Ag/a-TiO_2. (a) FESEM, (b) Ti, (c) O, (d) Cd, (e) S, (f) Ag.

Table S1. A summary of the element contents according to the XPS spectra.

Samples	Percent content of atom						
	Cd	S	Ti	O	Ag	C	N
CdS QDs	5.53	4.78	--	4.79	--	25.64	59.27
CdS/a-TiO_2	2.55	3.44	15.21	34.06		44.75	--
CdS/Ag/a-TiO_2	0.34	2.13	19.7	51.88	0.51	25.44	--
CdS/Ag/c-TiO_2	0.62	1.76	17.99	51.41	0.28	27.93	--

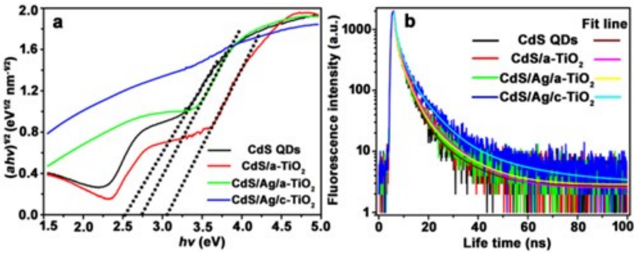

Fig. S4 (a) Tauc plots of as-prepared samples. (b) Time-resolved PL of as-prepared samples under the excitation of a pico-second (ps) 405 nm laser pulse.

The excited state electron radioactive decay lifetime of as-prepared samples were are shown in Fig. S5b, the calculated average lifetime of CdS QDs, CdS/a-TiO_2, CdS/Ag/a-TiO_2 and CdS/Ag/c-TiO_2 are 3.5, 4.4, 4.3 and 4.9 ns, respectively. The

increased lifetime of CdS/Ag/c-TiO_2 could be ascribed to the rapid carrier separation with a low recombination rate, which is in accordance with decreasing the PL peak intensity.[2, 3] Although the long-lived charge separated states make the CdS/Ag/c-TiO_2 more suitable for photocatalytic degradation of a variety of pollutants, it could be unsuitable for the selective photocatalytic oxidation of organic due to increase the effect of ·OH.

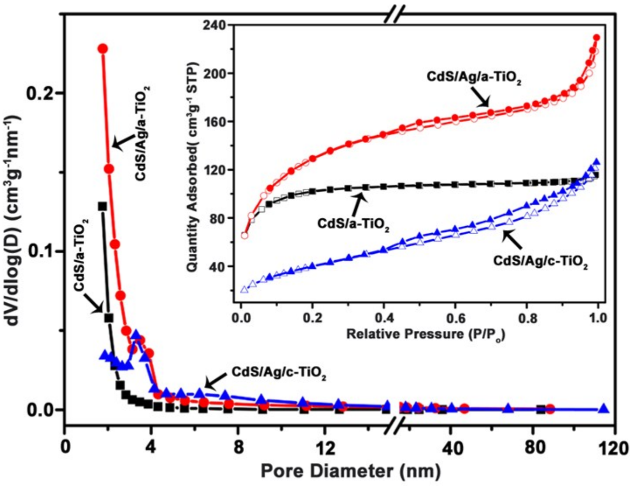

Fig. S5 Pore size distributions of as-prepared CdS/a-TiO_2, CdS/Ag/a-TiO_2 and CdS/Ag/c-TiO_2. The inset is N_2 adsorption–desorption isotherms.

Table S2. The BET surface area, pore volume and average pore size of the photocatalysts.

Samples	BET surface area [m^2 g^{-1}]	Total pore volume [cm^3 g^{-1}]	Average pore size [nm]
CdS/a-TiO_2	319	0.18	2.2
CdS/Ag/a-TiO_2	441	0.35	3.2
CdS/Ag/c-TiO_2	147	0.20	5.3

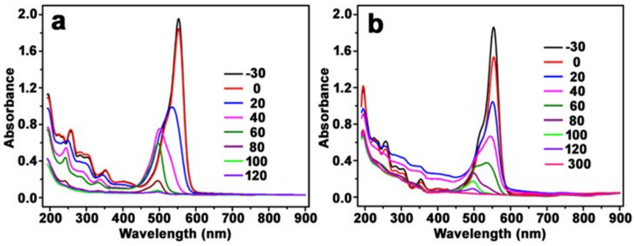

Fig. S6 UV−visible spectrum changes of RhB (10 mg L^{-1}) under blue LED lights. (a) Ag/a-TiO_2, (b) Ag/c-TiO_2. Three 1 W LED lights with a radiant wavelength of 455–460 nm were used as the light source. The characterization of Ag/a-TiO_2 and Ag/c-TiO_2 were reported in literature 3.

It is observed that RhB can be selectively oxidized respectively by unannealed Ag/TiO_2 (Ag/a-TiO_2, Fig. S6a) and annealed Ag/TiO_2 (Ag/c-TiO_2, Fig. S6b) to produce Rh-110 under light-emitting diode (LED) light (Three 1 W LED lights with a radiant wavelength of 455–460 nm were used as the light source). However, the conversion ratio of Ag/a-TiO_2 composites is higher (about 39% at 40 min) than that of Ag/c-TiO_2 composites (14% at 80 min) due to that the serious overoxidation of product was unavoidable.[4, 5]

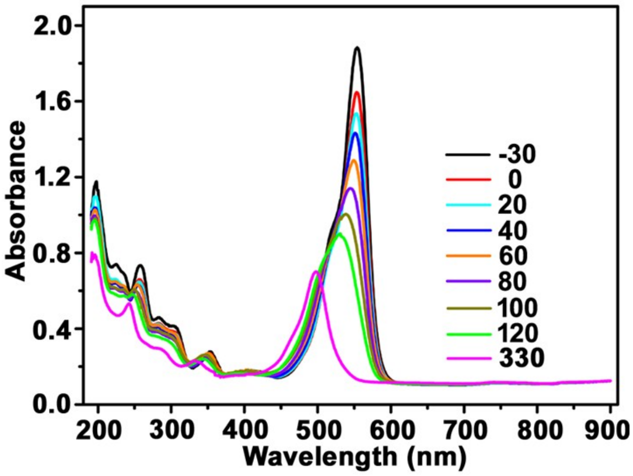

Fig. S7 UV−visible spectrum changes of RhB (10 mg L^{-1}) under UV-light irradiation when the CdS/a-TiO_2 is used as photocatalyst (2 g L^{-1}).

Reference

1. X. Wang, Z. Jia, F. Liu, H. Liang, X. You, K. Wang, X. Lou, W. Shuang, L. Xiao, B. Cai and L. Yang, *RSC Adv.*, 2016, **6**, 48985-48994.
2. X. Zhang, Y. Li, J. Zhao, S. Wang, Y. Li, H. Dai and X. Sun, *J. Power Sources*, 2014, **269**, 466-472.
3. J. Wang, D.-X. Ye, G.-H. Liang, J. Chang, J.-L. Kong and J.-Y. Chen, *J. Mater. Chem. B*, 2014, **2**, 4338.
4. B. Zhang, J. Li, Y. Gao, R. Chong, Z. Wang, L. Guo, X. Zhang and C. Li, *J. Catal.*, 2017, **345**, 96-103.
5. H. Liang, Z. Jia, H. Zhang, X. Wang and J. Wang, *Appl. Surf. Sci.*, 2017, **422**, 1-10.
